# Genome-Wide Association Study of Body Mass Index and Body Fat in Mexican-Mestizo Children

**DOI:** 10.3390/genes10110945

**Published:** 2019-11-19

**Authors:** Paula Costa-Urrutia, Valentina Colistro, Angélica Saraí Jiménez-Osorio, Helios Cárdenas-Hernández, Jacqueline Solares-Tlapechco, Miryam Ramirez-Alcántara, Julio Granados, Iván de Jesús Ascencio-Montiel, Martha Eunice Rodríguez-Arellano

**Affiliations:** 1 Laboratorio de Medicina Genómica, Hospital Regional Lic. Adolfo López Mateos, ISSSTE. 1321 Universidad Avenue, Álvaro Obregón, Florida, Mexico City P.C0103, Mexico; paula.costa.urrutia@gmail.com (P.C.-U.); jimenez.osorio.as@gmail.com (A.S.J.-O.); ibi_cardenashelios@yahoo.com.mx (H.C.-H.); jsoltlapechco@gmail.com (J.S.-T.); nutriologa.miryamra@gmail.com (M.R.-A.); 2 Departamento de Métodos Cuantitativos, Facultad de Medicina, Universidad de la República, 2125 General Flores Avenue, Montevideo P.C11800, Uruguay; valentinacolistro@gmail.com; 3 División de Inmunogenética, Departamento de Trasplantes, Instituto Nacional de Ciencias Médicas y Nutrición Salvador Zubirán. 15 Vasco de Quiroga Avenue. Mexico City P.C.14080, Mexico; julgrate@yahoo.com; 4 Coordinación de Vigilancia de Epidemiología, Instituto Mexicano de Seguro Social, 120 Mier y Pesado Street, del Valle Benito Juárez, Mexico City C.P. 03100 Mexico; ivan-ascencio@hotmail.com

**Keywords:** GWAS, BMI, percent of body fat, childhood

## Abstract

Background: Childhood obesity is a major health problem in Mexico. Obesity prevalence estimated by body mass index (BMI) is almost half than that estimated by percent body fat (%BF) in the Childhood Obesity pediatric cohort (COIPIS). Objective. We performed a genome-wide association study (GWAS) of BMI and %BF in 828 children from the COIPIS to identify markers of predisposition to high values for both phenotypes used for obesity classification. Methods: For the GWAS we used the LAT Axiom 1, Affymetrix and 2.5 million single loci from the 1000 Genomes Phase 3 imputation panel. We used a linear model, adjusted by age, sex, and Amerindian ancestry assuming an additive inheritance model. Results. Genome-wide significance (*p* ≤ 5.0 × 10^−8^) and 80% of statistical power was reached for associations of two loci in two genes (CERS3 and CYP2E1) to BMI. Also, 11 loci in six genes (ANKS1B, ARNTL2, KCNS3, LMNB1, SRGAP3, TRPC7) reached genome-wide significance for associations to %BF, though not 80% of statistical power. Discussion: None of the SNPs were previously reported as being associated to BMI or %BF. In addition, different loci were found for BMI and %BF. These results highlight the importance of gaining deeper understanding of genetic markers of predisposition to high values for the phenotypes used for obesity diagnosis.

## 1. Introduction

Childhood obesity is associated with severe health problems and premature death [1]. Mexico ranks as one of the first countries worldwide in childhood overweight/obesity with a mean national prevalence of 34.9% for school children [2]. Recently, for children from Mexico City in the pediatric Childhood Obesity cohort of the Healthy Childhood project (COIPIS) obesity prevalence estimated by percent body fat (%BF) was 43.7%, while it was 20.1% when estimated by body mass index (BMI) [3]. It means that the diagnosis of obesity by BMI underestimated around 50% of children diagnosed with obesity by %BF [3]. Deeper knowledge on the molecular etiology of the common phenotypes (or intermediate phenotypes) used for obesity diagnosis is key for improving its precision, and genome-wide association studies (GWAS) have been largely used for this purpose [4]. GWAS of BMI and/or obesity are typically performed in adults [4]; however, GWAS in children are recommended to identify what could affect early life [5]. In this regard, GWAS and meta-analysis in childhood populations with European ancestry have identified several of the novel loci [6,7]. Further, based on the 15 most strongly associated loci, it was found that the genetic predisposition to high BMI in childhood was associated with increased risk of type 2 diabetes and cardiovascular diseases in adult life [8].

In Mexico, the genetic components predisposing to high BMI and/or obesity in childhood are scarcely known. Efforts made have been concentrated in transferability studies from European adults to Mexican children, which showed partial BMI/obesity associated loci transferability [9,10,11,12,13,14], whereas a GWAS has not been reported in childhood population yet. To identify single nucleotide polymorphisms (SNPs) influencing BMI and %BF in Mexican children from COIPIS, we performed a GWAS using LAT Axiom 1, Affymetrix, and around 2.5 million SNPs from the 1000 Genomes Phase 3 imputation panel. 

## 2. Materials and Methods

This study includes a total of 828 Mexican-Mestizo children (423 boys, 405 girls) from Mexico City, aged from 3 to 16 years old, of the COIPIS from the Genomic Medicine Laboratory at Hospital Regional Lic. Adolfo López Mateos, Instituto de Seguridad y Servicios Sociales de los Trabajadores del Estado, ISSSTE (Institute of Security and Social Services of State workers) [3].

Weight, height, and %BF were measured using InBody J10 equipment (Gangnam-gu, Seoul 135–854 KOREA). Children were measured after a 10-hour fast, without consuming water, and they were barefoot and wearing light clothes. Accuracy of the stadiometer integrated to InBody J10 was ± 0.1 cm and ± 0.01 kg for height and weight, respectively. Electrical bioimpedance was used to estimate %BF as implemented in InBody J10 tetrapolar equipment of three frequencies (5, 50, and 250 kHz) and anthropometry [15]. BMI (kg/m^2^) was calculated as body weight (kg) divided by square height (m^2^). 

Genomic DNA was obtained from a whole blood sample. We used an automated system (QIASymphony, QIAGEN Hilden, Alemania). Genotyping was performed on Axiom^®^ Genome-Wide LAT 1 Array, designed for Latin American populations with 813,551 single nucleotide polymorphism (SNPs) at the Affymetrix Services Lab (California, USA, http://www.affymetrix.com/). Genotype calling was conducted using the Genotyping Console from Affymetrix (Axiom Analysis Suite). The SNP calling was done following Affymetrix best practice workflow, which includes the Genotyping Console Software in combination with SNPolisher. These pipelines include SNP and sample call rate thresholds, Dish QC filtering, and heterozygosity. No samples were discarded due to sex discordance. Regarding population structure, two individuals were considered outliers and were not included in downstream analysis.

In addition, we used 2.5 million of SNPs from HapMap European-ancestry imputation panel. Before imputation, we applied quality filters; SNPs with a minor allele frequency below 1% (MAF) and SNPs with poor imputation quality (<98%) were removed from the database (MACHr2_hat ≤ 0.3, IMPUTE proper_info ≤ 0.4orinfo ≤ 0.4). The Hardy–Weinberg equilibrium was estimated using Fisher’s exact test, and linkage disequilibrium among the SNPs was tested using the R^2^ coefficient. Both analyses were conducted in PLINK 1.9 software [16]. SNPs failing any of these filters were removed from downstream analyses. We performed imputation using Eagle software for phasing [17] and 1000 Genomes Phase 3 as a reference panel using the algorithm Minimac 3 [18]. We filtered out variants with MAF <1% and non bi-allelic variants. In order to assess imputation quality control, genotyped SNPs were masked in the imputation and MAF of imputed/genotyped SNPs were compared afterwards. Mean differences were <1%.

Population structure analysis was conducted using Principal Component Analysis (PCA) in Eigenstructure [19] and individual admixture proportion was obtained using Admixture software version 1.3 [20]. The top three significant PCs after Tracy-Widom test were included. Data from parental population of European, African, Asian, and Native American individuals were obtained from 1000 Genomes Project (http://www.internationalgenome.org/), while Amerindian genotypes were available at the server of the Project which collected the samples (ftp://ftp.inmegen.gob.mx/). GWAS of BMI and %BF were obtained using a linear model, adjusted by age, sex, and Amerindian ancestry (AMA) assuming additive inheritance model in PLINK 1.9 software [16]. Power calculations to detect significance at 5.0 × 10^−8^ were performed using a continuous outcome design in Quanto software version 1.2.4 (University of Southern California, Los Angeles, CA; (University of Southern California, Los Angeles, CA; http://biostats.usc.edu/Quanto.html). [21]. Calculations were carried out for gene only, under an additive inheritance model and using the minor SNPs allele frequency 0.02. The whole sample means and SD for BMI and %BF were used (BMI mean = 19.2, SD = 5.1; %BF mean = 28.5, SD = 11.3). Our study had 80% statistical power to detect β ≥ 10 with an allele risk frequency less than 0.02. As allele frequency increased from 0.02 β ≥ 3 could be detected.

For the genes with (or near) those SNPs reaching genome-wide significance (*p* ≤ 5.0 × 10^−8^) or significance at 5.0 × 10^−8^ ≤ *p*-value < 1.0 × 10^−7^passing Bonferroni correction (*p* < 0.05) we ran enrichment analyses for gene ontology using Enrichr web server software [22] to identify genes sharing a common biological function.

Parents of all children authorized their participation signing an informed consent. This project was approved by the Research and Ethics Committee of Regional Hospital Lic. Adolfo López Mateos (Registry number 447.2016) from ISSSTE.

## 3. Results

A total of 828 children (423 boys, 405 girls) aged from three to 16 were genotyped. The number of children, BMI and %BF means and standard deviation by sex and age are shown in Table 1. Distribution of BMI and %BF for the whole population is shown in Supplementary Materials Figure S1.

Regarding population structure, Mexican-Mestizo children formed a spread cluster distributed between the European, and Native-American parental populations—the expected pattern for Mestizo population [23,24]—with a mean of 57% (SD = 21%) of AMA and 36% (SD = 19%) of European ancestry. Population structure results are shown in Supplementary Materials Figure S2.

**Figure 1:**
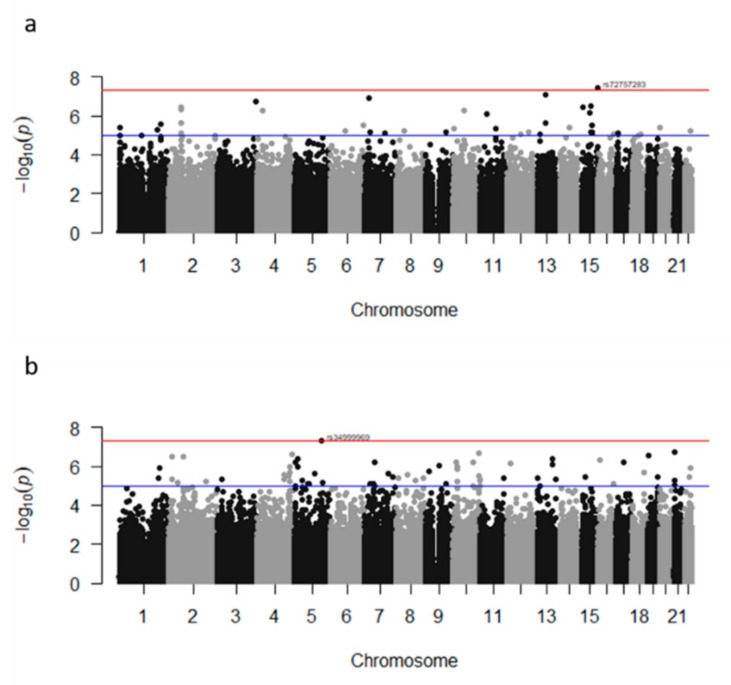
Manhattan plot of results of the genome-wide association study before imputation of (**a**) body mass index; and (**b**) percent body fat in children from Mexico City. Chromosomes are shown on the x-axis, and the −log_10_ of the *p*-value on the y-axis. The red line represents the genome-wide significance cut off of 5.0 × 10^−8^. The blue line shows false discovery rate significance cut off.

Overall, two loci in two genes reached genome-wide significance (*p* ≤ 5.0 × 10^−8^) associated with BMI (*CERS3*, *CYP2E1)* and 11 loci in 6 genes (*ANKS1B*, *ARNTL2*, *KCNS3*, *LMNB1*, *SRGAP3*, *TRPC7)* were associated to %BF. Before imputation, two loci reached genome-wide significance. One of them was associated to BMI (*CERS3* rs72757283) (Figure 1a) and the other was associated to %BF (rs34, 999,969 near *TRPC7*) (Figure 1b). After imputation, associations for both SNPs remained significant. Three additional SNPs in genes *PCDH15* and *CERS3* were associated to BMI, while six additional SNPs in genes, *ENAM*, *MARCH3*, *PHF20L1*, *SLC6A1*, *TRPC7,* and *ZC3H3* were associated to %BF at 5.0 × 10^−8^ < *p*-value < 1.0 × 10^−7^ significance level and passed Bonferroni correction (Table 2).

Variants in a single gene showed high Linkage Disequilibrium (LD), indicating that they correspond to the same signal. SNPs associated to BMI reached 80% of statistical power, whereas SNPs associated to %BF did not. Locus zoom plots of variants associated to BMI are shown in Figure S3.

Genes associated to BMI were enriched in drug catabolism, terpenoids, and ceramide metabolism, while those associated to %BF were enriched in transcription processes: regulation of mRNA and protein export from nucleus and ribonucleoprotein complex localization. The top five enriched terms are shown in Table 3. 

## 4. Discussion

Genome-wide significance (*p* ≤ 5.0 × 10^−8^) and 80% of statistical power was reached for associations of three loci in two genes (*CERS3* and *CYP2E1*) to BMI. Also, 11 loci in six genes (*ANKS1B, ARNTL2*, *KCNS3*, *LMNB1*, *SRGAP3*, *TRPC7*) reached genome-wide significance for associations to %BF, though not 80% of statistical power.

In this GWAS of childhood BMI and %BF conducted in 828 children (423 boys, 405 girls) aged from three to 16 years old, we found two loci in two genes associated to BMI, and 11 loci in or near six genes associated to %BF which reached genome-wide significance (Table 2). Three additional SNPs in genes *PCDH15* and *CERS3* were associated to BMI, and six additional SNPs in genes *ENAM, MARCH3, PHF20L1, SLC6A1, TRPC7,* and *ZC3H3* were associated to %BF at 5.0 × 10^−8^ ≤ *p*-value < 1.0 × 10^−7^ significance level and passed Bonferroni correction (Table 2). Overall, variants in a single gene showed high LD, indicating they correspond to the same signal. All loci associated to %BF did not reach 80% of statistical power; thus, caution is needed to evaluate findings related to %BF.

Neither the loci, nor the genes showing significant associations were previously reported as related to childhood BMI, %BF or obesity in Europeans. Mexico City, considered part of Central Mexico, has higher Amerindian ancestry than northern and western Mexico, [25]. In agreement with previous studies, on average, more than half of Mexican ancestry from Central Mexicans is Amerindian (mean AMA = 57% in this study) [26,27]. Ancestry differences could be explained, at least in part, by differences in genetic markers predisposing high childhood BMI and %BF values between Mexican and European children. Our results also agree with the findings of partial loci transferability from European adults to Mexican children; those studies found that 25 out of over 150 loci associated to BMI/obesity in Europeans were also associated in Mexican children [9,10,11,12,13,14].

Regarding genetic predisposition to high BMI and %BF values, and in line with previous results in adult Mexicans [28], different loci were associated with both phenotypes. This result may have more than one non-exclusive explanation. Firstly, even if the same several loci were involved in the genetic predisposition to high BMI and %BF levels, these loci could have different magnitude effect on each phenotype. Thus, the effect in both phenotypes could be detected by increasing the statistical power with a larger sample size [29]. This explanation could account for not finding replicates in other populations. Secondly, BMI and %BF are based on different anthropometric measurements and, in Mexican children, they reflect highly different obesity prevalences [3]. In particular, BMI by definition is a relationship between height and weight, but does not distinguish between fat and lean mass tissue [30]. A different feature of both phenotypes; BMI and %BF could be led by a different biological process, which was suggested by enrichment analysis (Table 3). In this regard, the genes associated with BMI (*CERS3*, *PCDH15*, *CYP2E1*) are involved in lipid and glucose metabolism, while the biological processes related to body fat metabolism of the remaining loci associated to %BF in genes *KCNS3*, *SRGAP3*, *SLC6A1*, *ENAM*, *LMNB1*, *MARCH3*, *TRPC7*, *PHF20L1*, *ZC3H3*, *ANKS1B* (Table 2) are not known. They were enriched in the transcription process (Table 3) in which small but important signals of regulation may drive cellular machines and trigger changes in metabolic genes but their involvement in fat metabolism remains uncertain [31]. In addition, loci associated to %BF did not reach the acceptable threshold of 80% of statistical power; thus, below we will focus our discussion on BMI associated SNPs.

The ceramide synthase 3 gene (*CERS3*) encodes for the ceramide synthase 3 protein; it is one of the six synthetases involved in de novo formation of ceramides [32,33]. Ceramides are the building blocks of sphingolipids, and their accumulation in tissues is involved in disorders associated with obesity. Although the cause is not fully understood, clinical studies have shown a positive correlation between plasma and tissue ceramide levels and insulin resistance [34]. In addition, after bariatric surgery in subjects with obesity, the decrease in adipose tissue is accompanied by a reduction in CERS3 products [35]. CERS3 is also found in immune system pathways. Moreover, the low-grade inflammation associated with obesity causes the infiltration of *TNFα* into adipocytes. The increase in this pro-inflammatory cytokine plus the increase in free fatty acids trigger de novo synthesis of ceramides [34].

The protocadherin-related 15 gene (PCDH15) is a member of the cadherin superfamily. Family members encode integral membrane proteins that mediate calcium-dependent cell-cell adhesion [36] Variants of this gene have been associated with triglycerides, total cholesterol, and apolipoprotein B levels in families with hyperlipidemia, suggesting their association with lipid abnormalities [37]. As well, other variants of this gene were associated with cardiovascular traits in Europeans [38] and with the carotid intima media thickness in Chinese population [39].

The cytochrome P450 family 2 subfamily E member 1 gene (*CYP2E1*) encodes the cytochrome P450 family 2 subfamily E member 1 enzymes; it is a potent protein of the oxidative system. It is involved in several preclinical and clinical lipid metabolism features. The *CYP2E* knockout mice showed protection against high-fat diet-induced obesity and insulin resistance, and also showed improvement in glucose homeostasis in vivo [40]. Regarding clinical studies, it was suggested that obesity increases *CYP2E1* activity in children [41].

This study has strengths and limitations that should be mentioned. The main limitation is sample size; 828 individuals is low in order to detect significant associations with low effects (β < 5 with an allele frequency of 0.02). The major strength is that the GWAS was conducted using intermediate phenotypes, BMI and %BF, widely employed for obesity classification, which gives us further insight in the etiology of the disease. 

## 5. Conclusions

In conclusion, we identified two loci in two genes (*CERS3* and *CYP2E1*) associated with childhood BMI which reached genome-wide significance and 80% of statistical power. These SNPs were not previously reported in population with European ancestry, probably due to ancestry differences or differences in magnitude effect of loci between Mexicans and Europeans. Our results highlight the mismatch of the genetic background predisposing high childhood BMI and %BF values. The BMI and %BF-related loci may reflect differences in different magnitude effect of associations, and/or different biological processes underlying these phenotypes. To our best knowledge, this is the first GWAS of BMI and %BF in Mexican children. Future efforts should include further replication studies to confirm the association of these potential loci to BMI and %BF in Mexican children.

## Figures and Tables

**Table 1:** Number (N), mean and standard deviation (SD) of body mass index (BMI) and percent body fat (%BF) by sex and age.

Age	N	BMI (SD)	%BF (SD)
Girls			
3	22	16.6 (1.7)	0.30 (0.13)
4	28	15.9 (1.4)	0.26 (0.14)
5	30	17.3 (3.4)	0.23 (0.15)
6	30	16.3 (1.7)	0.23 (0.04)
7	39	17.8 (3.6)	0.25 (0.13)
8	26	17.3 (3.0)	0.26 (0.08)
9	24	19.2 (2.6)	0.32 (0.07)
10	30	20.4 (3.8)	0.32 (0.10)
11	30	20.9 (3.9)	0.31(0.11)
12	28	22.0 (4.7)	0.32 (0.09)
13	34	22.1 (3.9)	0.33 (0.09)
14	37	23.8 (4.8)	0.37 (0.13)
15	23	23.0 (4.6)	0.33 (0.08)
16	24	25.7 (6.1)	0.33 (0.12)
Boys			
3	28	16.1 (2.2)	0.24 (0.16)
4	25	16.6 (2.9)	0.22 (0.13)
5	28	16.5 (2.6)	0.18 (0.13)
6	28	16.3 (2.0)	0.24 (0.11)
7	22	17.8 (2.3)	0.27 (0.12)
8	40	18.3 (3.1)	0.26 (0.10)
9	46	20.4 (4.2)	0.31(0.10)
10	29	22.6 (5.3)	0.34 (0.10)
11	32	21.6 (4.5)	0.30 (0.11)
12	41	21.2 (4.9)	0.28 (0.10)
13	28	22.3 (4.1)	0.27 (0.10)
14	36	21.9 (4.9)	0.24 (0.12)
15	20	23.6 (4.1)	0.26 (0.10)
16	20	25.5 (4.6)	0.27 (0.09)

**Table 2:** Significant loci associated with body mass index (BMI) and percent body fat (%BF) from the genome-wide association study after imputation analysis for Mexico City children. Reference SNP (rs), Ensemble identification (En Id), Chromosome (Chr), alleles (As), minor allele (MA), minor allele frequency (MAF), position (P) or function (F) in the gene (gene P/F), significance association level unadjusted *p*-value (Punadj), adjusted by Bonferroni (P_Bonf_). Genic upstream transcript variant (GUTV), genic downstream transcript variant (GDTV) and mean linkage disequilibrium among variants in the same chr (R^2^).

Gene	Description	rs	En Id	Chr	As	MA	MAF	P/G	β	Punadj	P_Bonf_	R^2^
BMI associated												
CERS3	Ceramide synthase 3	rs72757283	ENSG00000154227	15	A/C	C	0.02	intron	3.4	2.18 × 10^−8^	0.005	0.95
CERS3		rs75661572	ENSG00000154227	15	T/C	C	0.16	intron	1.3	8.97 × 10^−8*^	0.020	
CYP2E1	Cytochrome P450 family 2 subfamily E member 1	rs72866768	ENSG00000130649	10	A/G	G	0.02	intron	5.1	3.39 × 10^−8^	0.014	
PCDH15	Protocadherin related 15	rs2680328	ENSG00000150275	10	G/A	G	0.16	GUTV	1.4	5.86 × 10^−8*^	0.020	0.97
PCDH15		rs2799617	ENSG00000150275	10	G/A	G	0.16	GUTV	1.7	5.86 × 10^−8*^	0.024	
%BF associated												
ANKS1B	Ankyrin repeat and sterile alpha motif domain containing 1B	rs116928965	ENSG00000185046	12	G/A	A	0.130	3’ UTR	0.2	3.27 × 10^−8^	0.012	0.60
ARNTL2	Aryl hydrocarbon receptor nuclear translocator like 2	rs111392859	ENSG00000029153	12	G/A	A	0.012	intron	0.2	3.61 × 10^−8^	0.014	
ENAM	Enamelin	rs115766831	ENSG00000132464	4	T/A	A	0.014	3’ UTR	0.1	5.25 × 10^−8*^	0.031	
KCNS3	Potassium voltage-gated channel modifier subfamily S member 3	rs67939090	ENSG00000170745	2	A/T	T	0.012	intron	0.2	7.31 × 10^−11^	4.89 × 10^−5^	0.90
KCNS3		rs2198300	ENSG00000170745	2	A/G	G	0.012	intron	0.2	2.49 × 10^−11^	1.66 × 10^−5^	
KCNS3		rs111366249	ENSG00000170745	2	G/A	A	0.012	intron	0.2	3.67 × 10^−12^	2.45 × 10^−6^	
KCNS3		rs34364120	ENSG00000170745	2	C/A	A	0.012	intron	0.2	3.67 × 10^−12^	2.45 × 10^−6^	
KCNS3		rs13002427	ENSG00000170745	2	A/T	T	0.012	intron	0.2	1.26 × 10^−11^	8.40 × 10^−6^	
LMNB1	Lamin B1	rs140680370	ENSG00000113368	5	G/A	A	0.012	intron	0.2	1.40 × 10^−8^	0.007	0.57
MARCH3	Membrane associated ring-CH-type finger 3	rs77016412	ENSG00000173926	5	G/A	A	0.011	intron	0.2	9.17 × 10^−8*^	0.046	
PHF20L1	Phd finger protein 20 like 1	rs78211770	ENSG00000129292	8	A/G	G	0.050	intron	0.2	8.48 × 10^−9^	0.003	1
ZC3H3	Zinc finger CCCH-type containing 3	rs35110652	ENSG00000014164	8	C/T	C	0.015	GDTV	0.1	5.29 × 0^−8*^	0.023	
SLC6A1	Solute carrier family 6 member 1	rs58053962	ENSG00000157103	3	G/C	C	0.015	intron	0.2	6.64 × 10^−8*^	0.038	
SRGAP3	Slit-robo Rho GTPase activating protein 3	rs544274585	ENSG00000196220	3	C/A	A	0.015	intron	0.2	2.31 × 10^−8^	0.0133	
TRPC7	Transient receptor potential cation channel subfamily C member 7	rs3756699	ENSG00000069018	5	A/G	G	0.012	intron	0.1	3.53 × 10^−8^	0.0178	0.99
TRPC7		rs55996753	ENSG00000069018	5	G/A	A	0.050	intron	0.1	3.53 × 10^−8^	0.0178	
TRPC7		rs34999969	ENSG00000279240	5	C/A	A	0.050	near	0.1	7.18 × 10^−8*^	0.036	

*refers to loci with 5.0 × 10^−8^ ≤ *p*-value < 1.0 × 10^−7^ and passing Bonferroni correction.

**Table 3:** The top 10 significant enrichment terms in gene ontology (GO) biological processes of the genes associated with body mass index (BMI) and percent body fat (%BF) found in the genome-wide association study. Significance association level unadjusted *p*-value (P).

Biological Process	*P*
**BMI**	
Monoterpenoid metabolic process (GO:0016098)	9.0 × 10^−4^
Equilibrioception (GO:0050957)	9.0 × 10^−4^
Terpenoid metabolic process (GO:0006721)	1.0 × 10^−3^
Benzene-containing compound metabolic process (GO:0042537)	1.2 × 10^−3^
Neuromuscular process controlling balance (GO:0050885)	2.1 × 10^−3^
Epoxygenase P450 pathway (GO:0019373)	3.1 × 10^−3^
Exogenous drug catabolic process (GO:0042738)	3.4 × 10^−3^
Drug catabolic process (GO:0042737)	3.7 × 10^−3^
Ceramide biosynthetic process (GO:0046513)	4.9 × 10^−3^
Ceramide metabolic process (GO:0006672)	6.4 × 10^−3^
**%BF**	
Regulation of mRNA export from nucleus (GO:0010793)	3.3 × 10^−3^
Regulation of ribonucleoprotein complex localization (GO:2000197)	3.3 × 10^−3^
Manganese ion transport (GO:0006828)	4.9 × 10^−3^
Positive regulation of circadian rhythm (GO:0042753)	5.5 × 10^−3^
Poly(A) + mRNA export from nucleus (GO:0016973)	7.1 × 10^−3^
Regulation of RNA export from nucleus (GO:0046831)	8.2 × 10^−3^
mRNA polyadenylation (GO:0006378)	0.02
Transition metal ion transport (GO:0000041)	0.02
RNA polyadenylation (GO:0043631)	0.02
Regulation of protein export from nucleus (GO:0046825)	0.02

## Supplementary Materials

The following are available online at https://www.mdpi.com/2073-4425/10/11/945/s1, Figure S1: title Density distribution of percent body fat (%BF) and body mass index (BMI), Figure S2: title Genetic stratification of Mexican-Mestizo children from Mexico City., Figure S3: title Locus zoom plots of the GWAS after imputation with BMI.
