# Supplementary material for: Genome-Wide Association Study of Body Mass Index and Body Fat in Mexican-Mestizo Children
Source: Genes (Basel). 2019 Nov 19;10(11):945. doi: 10.3390/genes10110945 (PMC6895864; doi:10.3390/genes10110945)
Supplement: Supplementary file 1 [file genes-10-00945-s001.pdf]

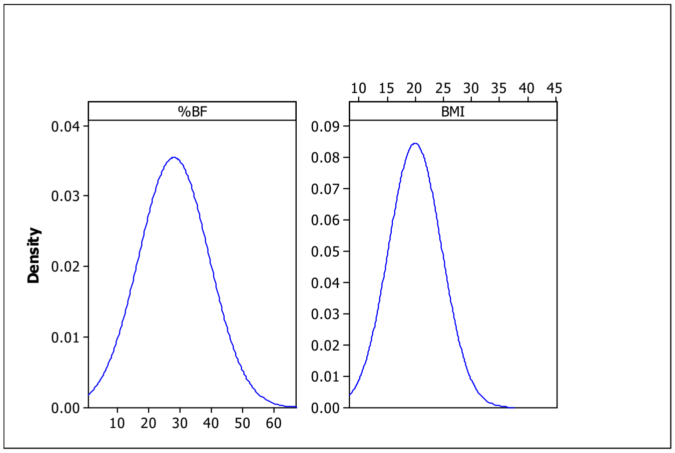


Figure S1. Density distribution of percent body fat (%BF) and body mass index (BMI). A total of 828 children (423 boys, 405 girls) aged from 3 to 16 from Mexico City of the pediatric Childhood Obesity cohort of the Healthy Childhood project (COIPIS) were used.

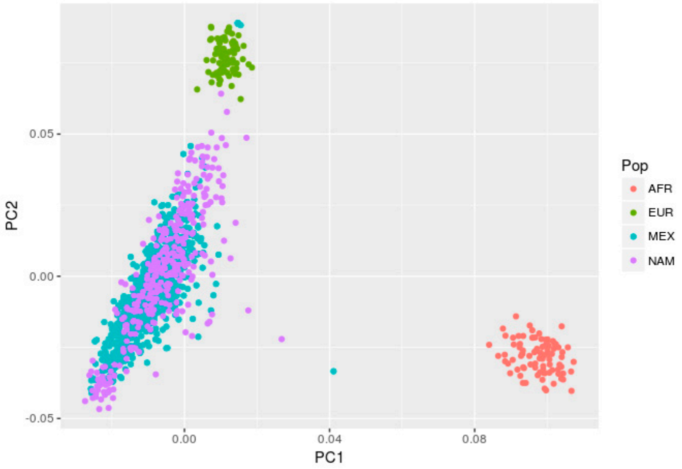


Figure S2. Genetic stratification of Mexican-Mestizo children from Mexico City. Principal Component Analysis shows the two most informative eigenvectors. Blue dots represent our Mexican children samples, Africans (AFR) are shown in red, Europeans (EUR) in green, and Mexican-Mestizo and Amerindian adults (NAM) in purple [1]. Two outliers in blue, above the European cluster, were eliminated for downstream analysis. Mexican-Mestizo children formed a spread cluster distributed between the European and Native-American parental populations. The overlap between our sample and that reported in Silva-Zolezzi et al. [1] confirmed the expected pattern for Mestizo population.

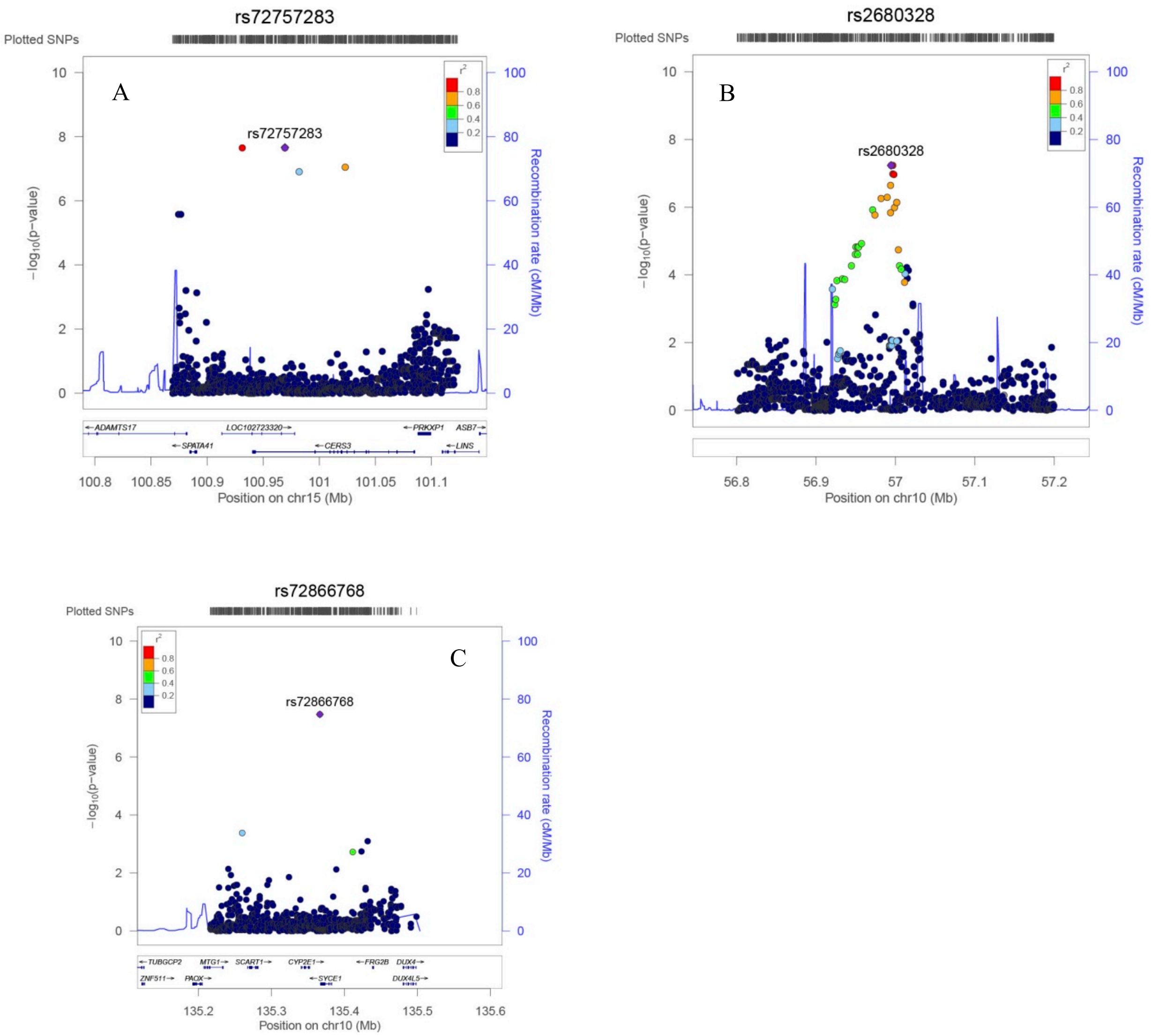


Figure S3. Locus zoom plots of the GWAS after imputation with BMI. Shading of the points represent the linkage disequilibrium (r2, based on the 1000 Genomes Project) between each SNP and the top SNP. (**A**) rs72757283 is in high LD with rs75661572 ($R^2$=0.95) in CERS3 gene. (**B**) rs2680328 is in high LD with rs2799617 ($R^2$=0.95) in PCDH15 gene. (**C**) rs72866768 in CYP2E1.

## References

1. Silva-Zolezzi I, Hidalgo-Miranda A, Estrada-Gil J, Fernandez-Lopez JC, Uribe-Figueroa L, Contreras A, et al. Analysis of genomic diversity in Mexican Mestizo populations to develop genomic medicine in Mexico. Proc Natl Acad Sci. 2009;106: 8611–8616. doi:10.1073/pnas.0903045106
